# Immunomodulatory effects of eubiotic and dysbiotic multi-species biofilms on oral keratinocytes

**DOI:** 10.1186/s12903-025-07576-w

**Published:** 2025-12-29

**Authors:** Madeleine Blomqvist, Martina Bardino Mørch, Bijar Ghafouri, Karin Wåhlén, Oonagh Shannon, Julia R. Davies

**Affiliations:** 1 Section for Oral Biology and Pathology, Faculty of Odontology , Malmö University, Malmö, SE 20506 Sweden; 2 Biofilms Research Center for Biointerfaces, Malmö University, Carl Gustavs väg 34, Malmö, SE 205 06 Sweden; 3 Department of Health, Medicine and Caring Sciences, Pain and Rehabilitation Centre, Linköping University, Linköping, SE 581 83 Sweden

**Keywords:** Bacterial consortium, Oral mucosa, Eubiosis, Dysbiosis, Inflammation

## Abstract

**Background:**

Elucidating host‒microbe interactions is essential for understanding oral health and disease. In periodontitis, the host inflammatory response to accumulated plaque shifts eubiotic biofilm communities toward dysbiosis, with enrichment of proteolytic bacterial species. The first line of host defence in the subgingival niche involves oral keratinocytes, which communicate with immune cells in the mucosa. Host responses to individual bacterial species have been widely characterized, but in this study, we used a co-culture model to better understand how changes in the multispecies biofilm phenotype affect keratinocyte effector function as well as the effects on inflammatory cells.

**Methods:**

Biofilms representative of eubiotic (HA) or dysbiotic (DA) bacterial communities were developed on nitro-cellulose membranes over 7 days and then co-cultured with oral keratinocytes for 6 h. Biofilm proteolytic activity was measured with a fluorescent substrate. Multiplex cytokine analysis of the co-culture medium was used to study keratinocyte responses and the activation of inflammatory cells was investigated via flow cytometry.

**Results:**

Proteolytic activity was greater in the DA biofilms than in the HA biofilms, most likely due to gingipains from *Porphyromonas gingivalis*. Keratinocytes released a range of cytokines, chemokines and growth factors, and the response to DA was more pro-inflammatory than that to HA biofilms, with relatively high levels of factors such as MIP-3*a*, IL-8, GM-CSF and IL-17 C. Co-culture medium from both the HA and DA biofilms elicited strong monocyte and neutrophil activation responses, although the effect of the DA biofilms was greater for monocytes than for neutrophils.

**Conclusions:**

In this study, we show that keratinocytes have distinct response profiles to HA as compared to DA periodontal biofilm communities. The combination of products from the biofilm and the activated keratinocytes generated significant activation of inflammatory cells. This in vitro model thus provides insight on the complex host-microbiome interactions during development of periodontal disease.

**Supplementary Information:**

The online version contains supplementary material available at 10.1186/s12903-025-07576-w.

## Introduction

The oral microbiome is one of the most diverse in the human body, with an estimated 200–300 different bacterial species present in a single individual [1]. The stationary element is found as microbial biofilms attached to teeth and soft tissue surfaces, with a mixture of free-floating bacteria shed from these biofilms present in saliva. The formation of complex, multispecies biofilms or dental plaque is initiated through interactions between bacteria expressing relevant receptors and ligands exposed in the surface pellicle formed by adhered saliva proteins [2]. The adhesion of “initial colonizers” allows coaggregation of additional species as well as growth and succession to form mature biofilms [3]. The synthesis of components by members of the microbial community, as well as the sequestration of host proteins, gives rise to a biofilm matrix that protects the growing communities [4] and facilitates interspecies communication and cross-feeding [5]. The composition of oral biofilms is niche-specific, reflecting local ecological determinants such as the availability of metabolic substrates and oxygen tension, processes occurring within the community, and the health status of the host [6].

In health, a balanced relationship, termed eubiosis, exists between biofilm communities and the host. Under these conditions, supragingival multispecies oral biofilms are usually dominated by species of *Streptococcus*, with *Veillonella*,* Actinomyces* and *Capnocytophaga* [7]. The keratinocyte barrier of the sulcus epithelium constantly interacts with microbial communities and controlled inflammation maintains immune homeostasis. Increases in the microbial load due to the build-up of plaque at the gingival margins initiate gingivitis, a reversible inflammatory reaction in periodontal tissues leading to increased flow of protein-rich crevicular fluid and patrolling neutrophils from the gingival sulcus. As the biofilm develops in the subgingival environment, perturbations in the local mileau can affect bacterial behaviour and change the composition towards a dysbiotic phenotype, which predisposes to disease [8, 9]. Increased availability of protein promotes growth of a range of proteolytic species [10] belonging to the red and orange complexes described by Socransky [11]. These include anaerobic bacteria *e.g. Fusobacterium nucleatum*, *Parvimonas micra* and *Porphyromonas gingivalis* as well as facultative anaerobic species such as *Streptococcus constellatus* [11, 12]. The combination of bacterial attributes and proinflammatory cytokine production can lead to progressively destructive non-resolving inflammation, culminating in degradation of the supporting periodontal tissues; periodontitis [13]. All the species mentioned above have been identified at increased relative abundances in periodontitis [14]. Despite our increasingly sophisticated understanding of the processes occurring within microbial communities, many aspects of how these changes are mirrored in the host response during the transition from health to periodontal disease remain poorly understood.

Oral keratinocytes offer the first line of defence at the oral mucosal barrier, responding to biofilms that develop in the subgingival niche and communicating with immune cells in the mucosa [15]. As such, they express pattern recognition receptors, including toll-like receptors (TLRs) and protease-activated receptors (PARs) [16]. In response to the sensing of bacterial virulence factors, the release of pro-inflammatory cytokines facilitates the recruitment and activation of inflammatory cells, most notably neutrophils and monocytes. Many studies have investigated the role of virulence factors (lipopolysaccharide, toxins and proteases) released from individual periodontal pathogens in mono-species culture on the secretion of pro-inflammatory cytokines from keratinocytes [17]. The importance of multi-species interactions in periodontitis has been emphasized [18, 19], and recently developed models that better encompass the complexity of the in vivo situation have been fundamental to advancing the understanding of the disease process [20–22]. In this study, we established multi-species communities of bacteria growing in biofilms that model health- or disease-associated communities and determined how they affect keratinocyte effector function as well as the downstream effects of co-culture medium on neutrophil and monocyte activation in the acute reaction to the presence of bacteria.

## Materials and methods

### Cell culture conditions

**Fig. 1:**
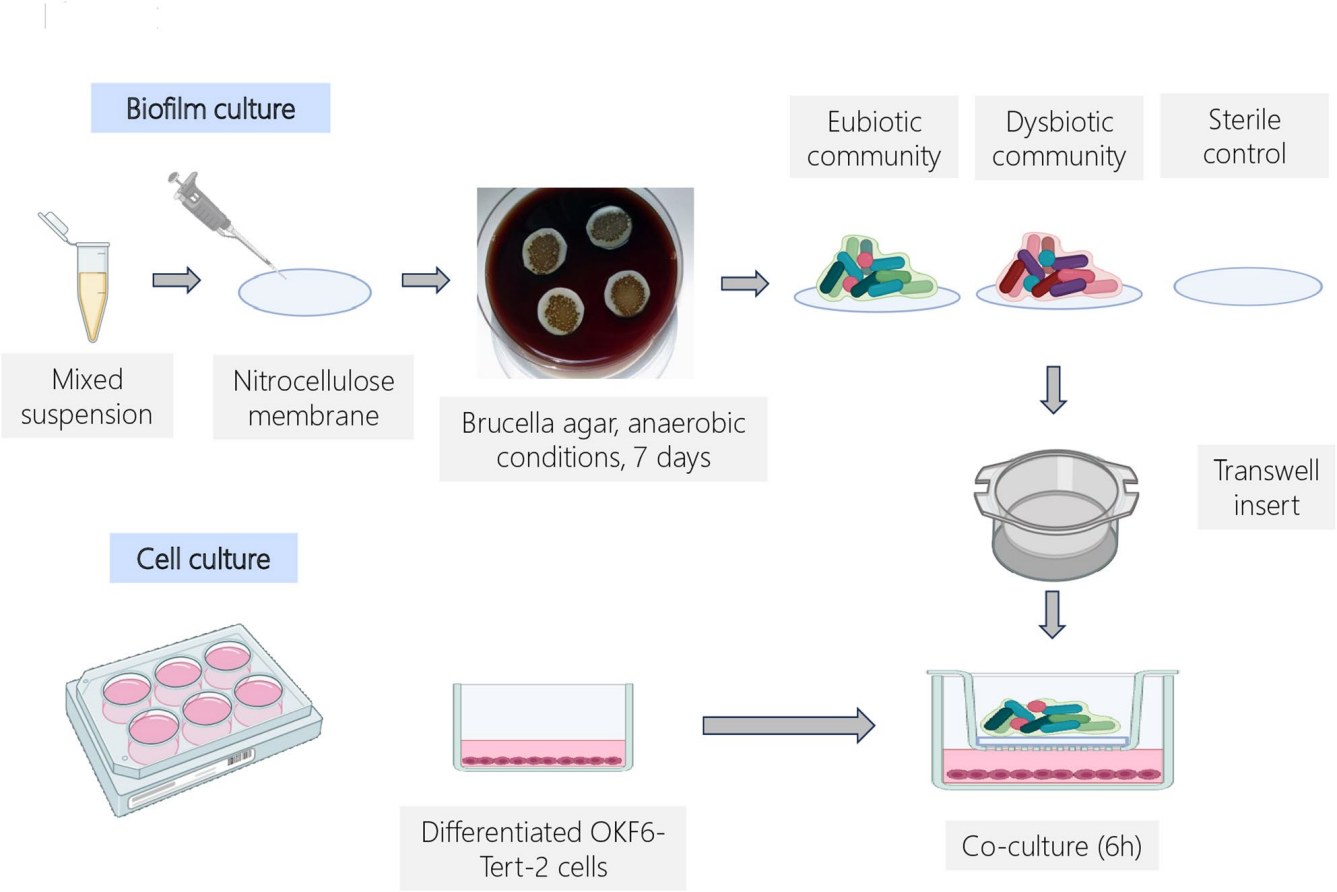
Schematic diagram showing the experimental set-up used for the co-culture experiments. Differentiated oral keratinocyte cell layers and multi-species biofilms were prepared separately before being co-cultured for 6 h. The experimental details are provided in the Materials and Methods. All experiments were performed in triplicate using independent bacterial and cell cultures

Immortalized normal human oral keratinocytes (OKF6/TERT-2) [23], a kind gift from Dr. James Rheinwald, Brigham and Women’s Hospital, Boston, USA, were used between passages 30 and 35. The cells were seeded into 6-well tissue culture plates and maintained in keratinocyte serum-free medium (Thermo Fisher Scientific, Paisley, UK) supplemented with 0.2 ng/ml human recombinant EGF, 25 µg/ml bovine pituitary extract and 0.3 mM CaCl_2_ containing 1 IU/ml penicillin and 1 µg/ml streptomycin (DF-K medium) until they reached 25% confluence (24–48 h). The cells were then grown to confluence in high-density medium (HDM) consisting of equal volumes of DF-K medium and a mixture of calcium- and glutamine-free media, Dulbecco’s modified Eagle’s medium (DMEM) and Hams F-12 at a 1:1 ratio (vol/vol) supplemented with 25 µg/ml bovine pituitary extract, 0.3 mM L-glutamine, 0.2 ng/ml human recombinant EGF, 1 IU/ml penicillin and 1 µg/ml streptomycin (Fig. 1).

### Bacterial culture conditions and preparation of HA and DA biofilms

The oral bacterial strains used in this study were chosen to model a eubiotic, health-associated (HA) consortium [*Streptococcus oralis* (89 C), *Streptococcus gordonii* (JD13A), *Streptococcus intermedius* (31B: D), *Shaalia odontolytica* G3H), *Veillonella parvula* (10BB) and *Capnocytophaga sputigena* (3B)] and a dysbiotic, disease-associated (DA) consortium [*P. micra* (EME), *F. nucleatum* (BK:0), *Streptococcus constellatus* (NCTC10714) and *P. gingivalis* (W50)] based on their enrichment in health and periodontitis respectively [11,12,14]. All strains, except the type strains of *S. constellatus* and *P. gingivalis*, were archived clinical isolates from the oral cavity. Strains maintained at -80 °C in skim milk were recovered on blood or Brucella agar (*P. gingivalis* and *F. nucleatum*) and allowed to grow for 4‒6 days at 37 °C in an atmosphere of 5% CO_2_, 10% H_2_ and 85% N_2_ (anaerobic conditions). A 1-µL loop of each strain was then recovered from the agar plates and suspended in 1 mL of pre-reduced brain‒heart infusion (BHI) medium to obtain an OD_600_ of approximately 1. Thereafter, 100 µl of each suspension was mixed to prepare each consortium. Fifty microliters of the consortium mixture were then spotted onto sterile nitro-cellulose membranes (diameter 2 cm) placed onto Brucella agar plates and maintained for 5–7 days under anaerobic conditions. See Fig. 1 for a schematic representation of the in vitro model.

### Co-culture of keratinocytes and biofilms

Co-cultures were carried out essentially as described by Bengtsson et al. [24]. Briefly, the HDM was replaced with a mixture of DMEM and Hams F-12 at a 1:1 ratio, and the level was adjusted to ensure that it would be just in contact with the underside of the insert, allowing the diffusion of products between the biofilm and the keratinocytes. The nitro-cellulose membranes with the established HA and DA biofilms were then placed on the mesh of sterile Transwell inserts and added to 6-well culture plates containing confluent keratinocyte cell layers for 6 h. This time point was established in pilot experiments and was chosen to reflect early innate responses while also avoiding toxic effects of the bacterial products on the cells. A sterile nitro-cellulose membrane without biofilm was used as a control (Fig. 1). To assess viability after co-culture, the cells were observed at 40x magnification under a Nikon TS100 Eclipse inverted microscope.

### Proteolytic activity

The general proteolytic activity of the co-culture medium was determined by mixing 10 µl aliquots with 100 µl of FITC-conjugated gelatin (1 mg/ml DQ™ gelatin, Molecular Probes) in a 96-well plate and incubating at 37 °C. The fluorescence was measured after 60 min using a BMG Clariostar plate reader (excitation 485 nm, emission 530 nm). The values for the negative control (cell medium alone) were subtracted, and the final values were expressed as trypsin equivalent units after comparison with a standard curve. A solution of 0.5 µg/ml porcine pancreatic trypsin (Sigma) was used as a positive control. Gingipain activity was measured by mixing 50 µl aliquots with 5 µl of a selective fluorescent substrate, 800 µM BikKam-16 [25] and incubating at 37 °C. Fluorescence was measured after 10 min as described above. Purified R gingipain (0.2 µg/ml, GingisREX, Genovis, Sweden) was used as a positive control.

### Keratinocyte cytokine secretome

After the experiment, the co-culture medium was harvested and frozen at -80 °C prior to cytokine analysis, which was carried out via the commercially available U-PLEX Biomarker Group 1 (hu) 71-plex (Meso Scale Discovery (MSD), Maryland, USA) panel. The panel contained cytokines, chemokines and growth factors. The assay was run according to the protocol provided and read on an MSD instrument Quickplex SQ120. The data were processed in Discovery Workbench version 4.0.12, where the light intensity of each protein‒antibody complex was converted into a concentration (pg/ml) via an 8-point standard curve of known protein concentrations. As a quality control, only the markers that were detected in at least 50% of the samples in the data analyses were included.

### Leucocyte activation in the presence of co-culture medium

Whole blood was collected from 7 healthy donors. The recruitment of healthy donors was approved by the National Ethical Review Authority (approval number 2023–03757). Monocyte and neutrophil activation in response to the co-culture medium was investigated via flow cytometry. Blood was incubated with co-culture medium in a volume that resulted in a ratio of 10:1 of blood: stimulant. HEPES buffer was used to determine background activation. The leukocyte agonist N-formyl-methionine-leucyl-phenylalanine (fMLF) (1 µM; Sigma‒Aldrich) was used as a positive control for neutrophil and monocyte activation. After stimulation, the samples were incubated for 15 min in the dark with markers of leucocyte activation in two panels: (a) anti-CD14-PE, anti-HLA-DR-FITC, anti-CD11b-Pe-Cy5, (b) anti-CD14-PE, anti-CD62L-Pe-Cy5, and anti-CD66b-FITC (all 1:10 from BD Biosciences). The samples were run on an Accuri C6 Plus flow cytometer (BD Biosciences), and the data were analysed via C6 Plus software. The monocytes were gated as a CD14-positive population. After excluding the monocytes, the neutrophils were gated as a weak CD14-positive population with high granularity (Supplementary Fig. 1). The CD11b, CD62L, HLA-DR, and CD66b intensities within these gates were analysed in histograms.

### Statistical analysis

All experiments were carried out at least three times with independent biological replicates. Proteolytic activity data were analysed via the nonparametric Kruskal‒Wallis test with Dunn’s test for multiple comparisons in GraphPad Prism v10.2.3. Flow cytometry data were analysed via the parametric paired t-test. Multivariate data analysis was performed via SIMCA version 18.0 (Sartorius Stedim Data Analytics AB, Umeå, Sweden) according to the methodology described by Wheelock & Wheelock [26]. Principal component analysis (PCA), an unsupervised analysis, was performed first to investigate any trends in the observations and to detect outliers. The outliers were identified via two methods: score plots in combination with Hotelling’s T2 (strong outliers) and distance to the model in X-space (moderate outliers). Orthogonal partial least square discriminant analysis (OPLS-DA) was performed to investigate the multivariate differences between the secretome and the composition of the biofilm. The model was considered to be significant if the p value for CV-ANOVA (cross-validated analysis of variance) was < 0.05 together with values of R^2^ and Q^2^. R^2^ describes the goodness of fit—the fraction of the sum of squares of all the variables explained by a principal component. Q^2^ describes the goodness of prediction, which is the fraction of the total variation of the variables that can be predicted via principal component cross-validation methods. The R^2^ value should be greater than Q^2,^ and the differences should not be greater than 0.3. Proteins with VIP (variable of importance) or VIP_pred_>1 and absolute p(corr) > 0.3 were considered to be significant. p(corr) is the loading of each variable scaled as a correlation coefficient (ranging from − 1 to + 1). All these parameters are reported for each analysis in the Results section.

### Network analysis

Protein-protein association networks and functions for the important proteins that differentiated the groups, were analysed using the online database tool Search Tool for Retrieval of Interacting Genes/Proteins (STRING, version 11) [27]. The protein names (multiple proteins) were entered in the search engine with the following parameters: organism = *Homo sapiens*; maximum number of interactions = query proteins only; interaction score = minimum required interaction score of high confidence (0.700) and an FDR ≤ 0.05 was used when classifying the Biological Process (GO) of each protein. In the network figure, each protein is represented by a coloured node, and protein-protein interaction and association are represented by an edge visualized as a line. Higher combined confidence scores are represented by thicker lines/edges.

## Results

### Composition of multispecies biofilms

**Fig. 2:**
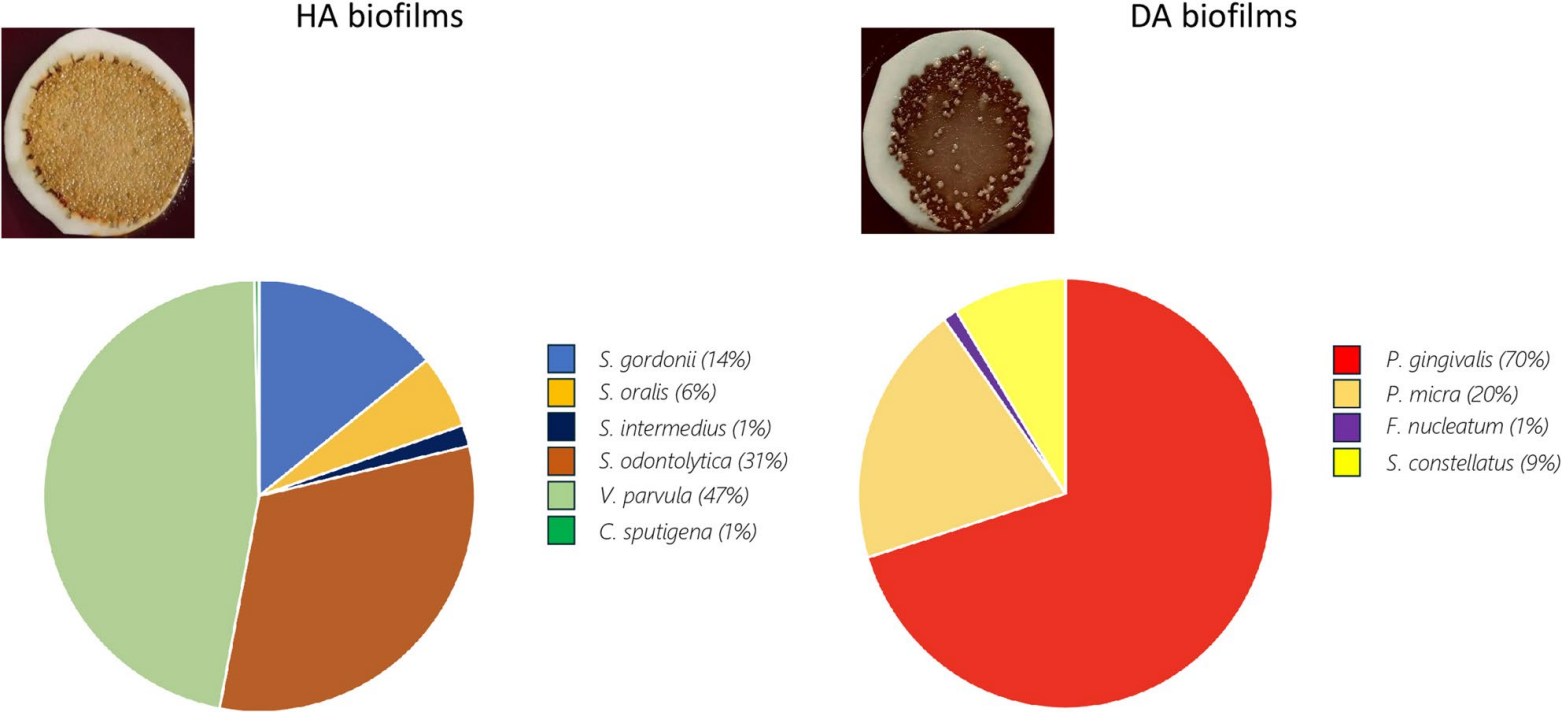
Pie charts showing the composition of representative eubiotic (HA) and dysbiotic (DA) biofilms cultured for 7 days on nitro-cellulose membranes placed on Brucella agar at 37 °C in 5% CO_2_, 10% H_2_ and 85% N_2_ and used for co-culture with oral keratinocytes

The composition of the HA and DA biofilms after 7 days of growth was determined by culturing a sample on Brucella agar, with differences in colony morphology as well as Gram staining used to distinguish between the species. This protocol for bacterial identification has previously been validated by using MALDI-TOF. The results demonstrated that all the species added to the initial inoculum were maintained and viable in both the HA and the DA biofilms (Fig. 2). Four independent experiments were performed that all gave similar relative proportions of bacterial species (± 5% of the values shown) within the consortia. In all cases the three dominant species in the HA biofilm were *V. parvula*, *S. odontolytica* and *S. gordonii*, while the relative amount of *C. sputigena* was very low. In the DA biofilm, growth on Brucella agar appeared to favour *P. gingivalis* and *P. micra* at the expense of *F. nucleatum* and *S. constellatus*.

### Proteolytic activity

**Fig. 3:**
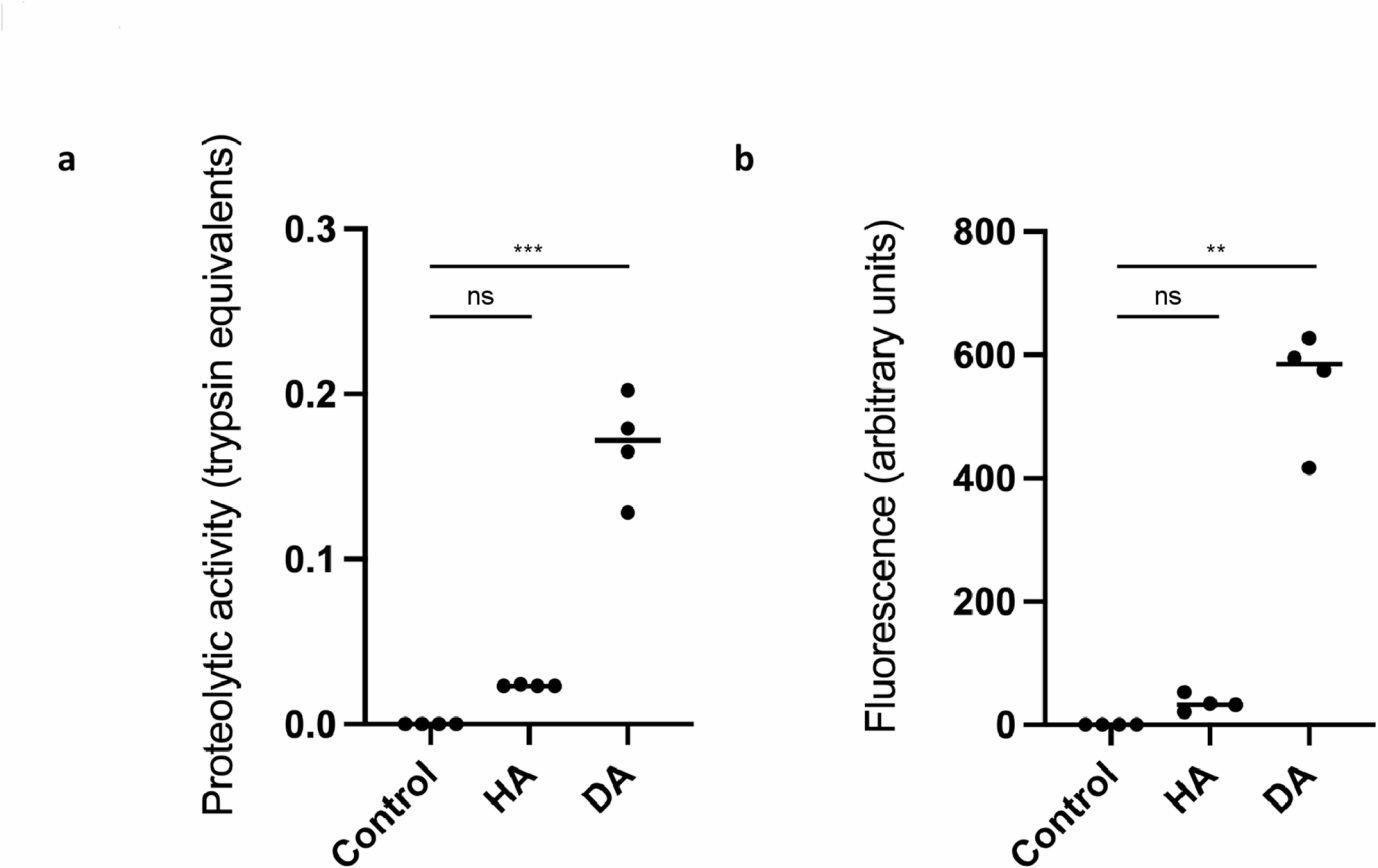
Proteolytic activity in cell supernatants after co-culture with control, health-associated (HA) or disease-associated (DA) biofilms for 6 h. **a** General proteolytic activity was measured using a FITC-conjugated gelatin substrate, with cleavage measured as fluorescence at 530 nm. The values were converted to trypsin equivalent units by comparison with a standard curve. (**b**) Gingipain activity was measured as above using BikKam-16 as a substrate, and values are expressed in arbitrary units. Statistical analysis was performed using the nonparametric Kruskal‒Wallis test with Dunn’s test for multiple comparisons in GraphPad Prism v10.2.3; ns = not significant, ** *P* < 0.01, *** *P* < 0.001

The proteolytic activity of the HA and DA biofilms was investigated using general and gingipain-specific fluorescent substrates. This showed that the co-culture medium from the HA biofilm contained low general proteolytic activity equivalent to that of the control (membrane with no biofilm), whereas the DA biofilm contained significantly higher levels of activity (*p* < 0.001) (Fig. 3a). As expected, gingipain activity was present at a significantly greater concentration in supernatants from the DA biofilms where *P. gingivalis* was present than in those from the HA biofilm or the control (*p* < 0.01) (Fig. 3b).

### Cytokine secretome of challenged oral keratinocytes

**Fig. 4:**
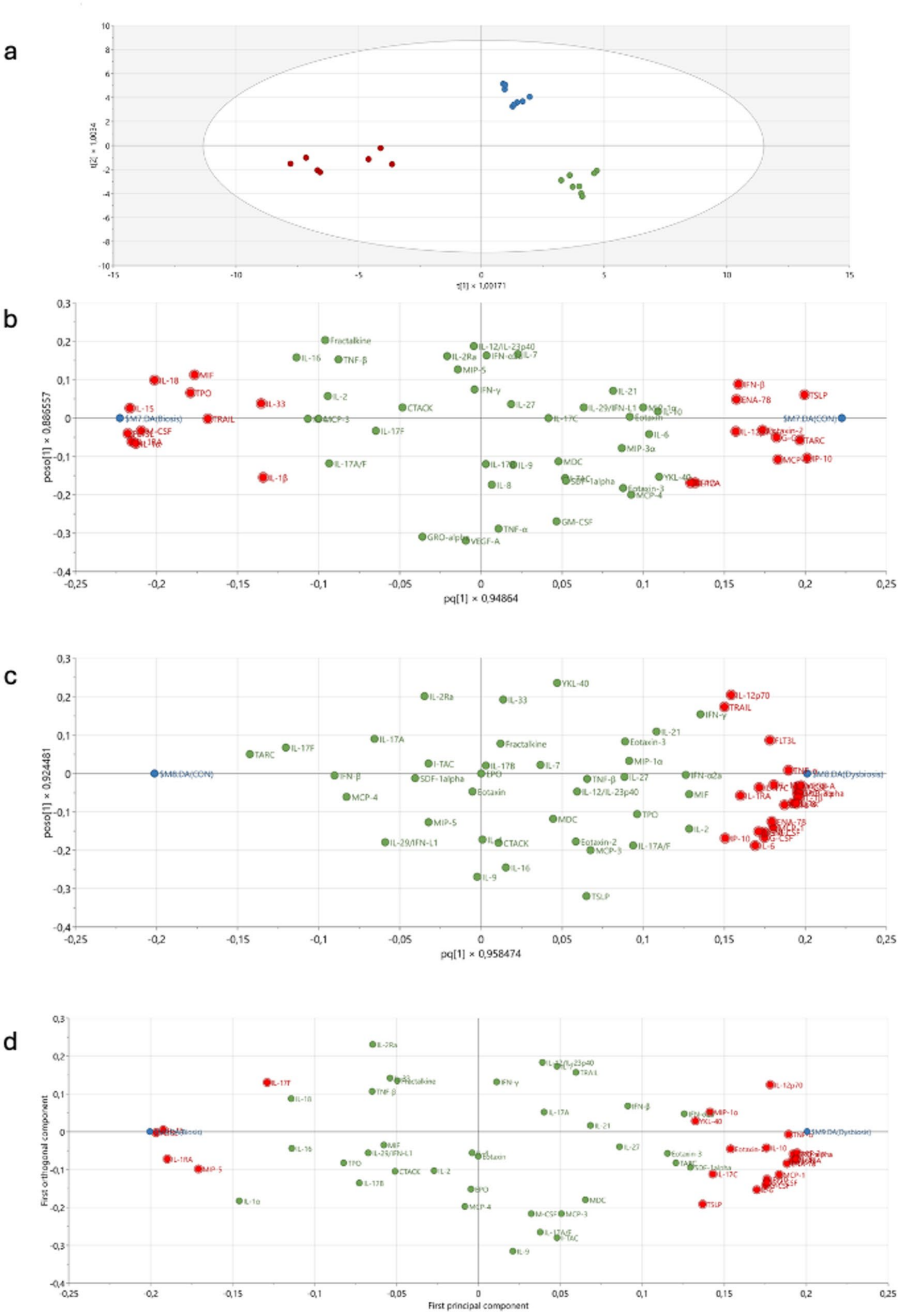
Release of immune mediators from activated keratinocytes. Cytokine, chemokine and growth factor levels were determined in the co-culture supernatants via a U-PLEX biomarker assay. Figure 4**a** shows a score plot of orthogonal partial least square discriminant analysis (OPLS-DA) comparing the groups: control (CON) – no biofilm (blue circles) as well as the health-associated (HA) (green circles) and disease-associated (DA) (red circles) biofilms. Figure 4**b** shows the loading plot for the OPLS-DA comparing HA and CON. Figure 4**c** shows the loading plot for OPLS-DA comparing DA and CON, and Fig. 4**d** shows the loading plot for OPLS-DA comparing HA and DA. The significant proteins (VIP > 1 and absolute p(corr) > 0.3) are marked in red in all loading plots

**Fig. 5:**
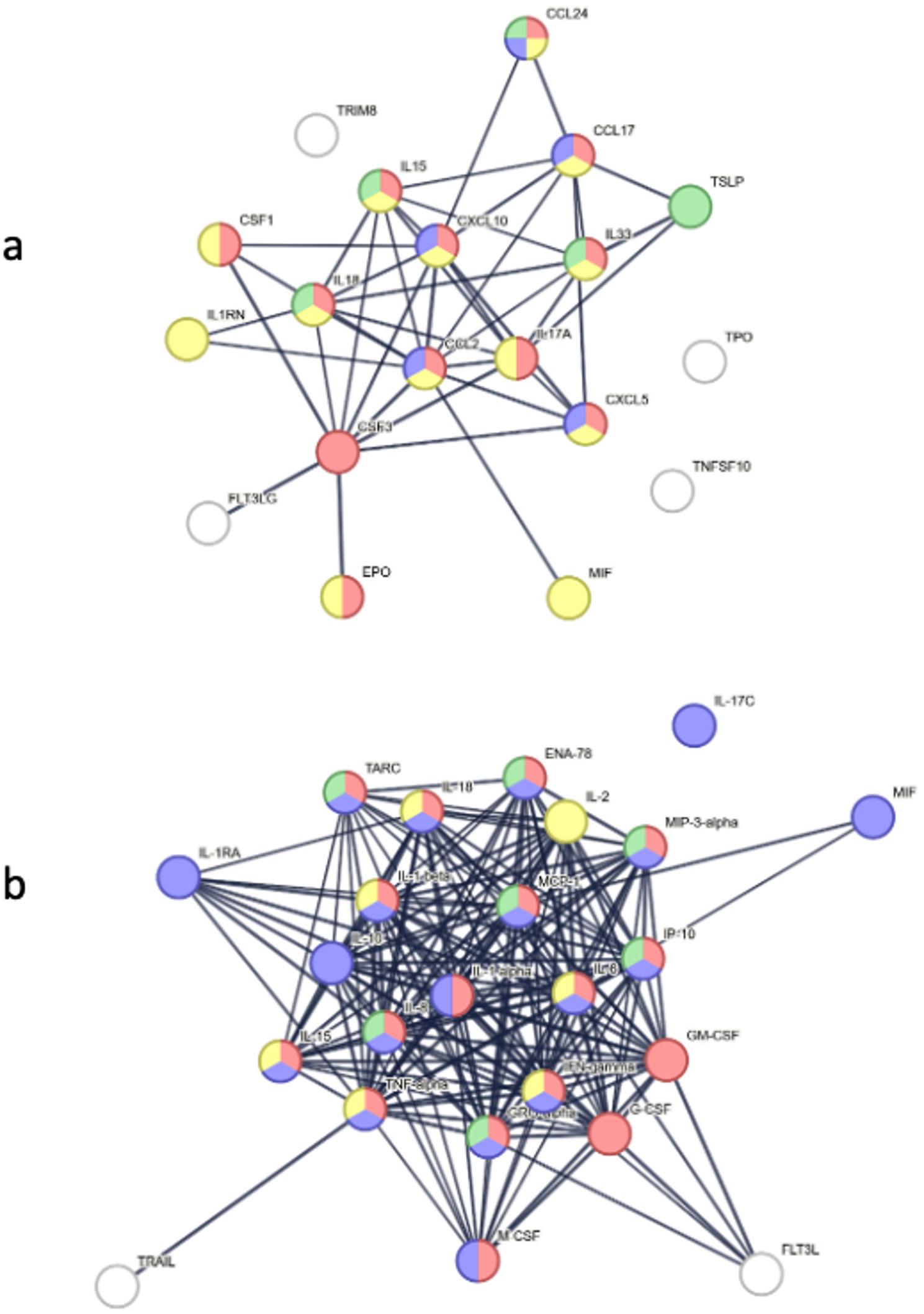
Network analysis of the significant proteins that discriminate the secretome of keratinocytes exposed to control, health-associated (HA) or disease-associated (DA) biofilms, from Tables 1 and 2 analysed using the STRING-protein database. Figure 5**a** shows proteins enriched in the HA group compared to control and Fig. 5**b** shows proteins enriched in the DA group compared to control. Each protein is represented by a colored node and protein-protein interactions are represented by lines, with higher combined confidence scores represented by thicker lines. Red and green circles indicate functions in cytokine-mediated and chemokine-mediated signaling pathways respectively, while blue circles indicate functions related to the inflammatory response and yellow circles show proteins involved in positive regulation of the inflammatory response

The cytokine secretome profiles of keratinocytes exposed to HA and DA biofilms were assessed via multiplex analysis against a panel of 71 cytokines/chemokines. The PCA score plots did not identify any outliers. The OPLS-DA model (two predictive components, R^2^ = 0.95, Q^2^ = 0.94, CV-ANOVA *p* = 6.89 × 10^− 20^) clearly demonstrated separation between the secretome from keratinocytes exposed to the control, the HA and the DA biofilm (Fig. 4a). The replicates from individual experiments were well clustered for each of the conditions. To identify proteins that contributed to the separation between the 3 groups, three different OPLS-DA models were used: HA-CON, DA-CON and HA-DA (Fig. 4b, c and d). The model for HA-CON (two components, R^2^ = 0.99, Q^2^ = 0.98, CV-ANOVA *p* = 4.65 × 10^− 09^) identified 22 proteins that significantly [VIP_pred_>1.0 and p(corr) > 0.3] contributed to the separation (Table 1). The DA-CON model (two components, R^2^ = 0.99, Q^2^ = 0.97, CV-ANOVA *p* = 1.58 × 10^− 07^) identified 27 proteins that significantly [VIP_pred_>1.0 and p(corr) > 0.4] discriminated DA from CON (Table 2). The OPLS-DA model for comparison between HA and DA (two components, R^2^ = 0.99, Q^2^ = 0.97, CV-ANOVA *p* = 8.28 × 10^− 08^) identified 27 significantly [VIP_pred_>1.0 and p(corr) > 0.3] altered proteins (Table 3). The concentrations of immune surveillance mediator, IL-15, pro-inflammatory mediator IL-1a and anti-inflammatory IL-1RA were greater in HA than in both DA and CON (Tables 1 and 3). The concentrations of 26 of the 27 proteins identified as significant were found to be greater in co-culture medium from cells exposed to the DA than in that from the CON group (Table 2). In particular, pro-inflammatory mediators MIP-3, IL-8, GM-CSF and IL-17 C had fold changes > 10 in keratinocytes exposed to DA biofilms. The differences in concentration were also found to be significant (*p* < 0.05) for the majority of cytokines according to Mann‒Whitney analysis, indicating that the proteins constitute some of the key differences observed between the groups (Tables 1, 2 and 3). STRING analysis was used to generate a functional overview of the secretome from keratinocytes exposed to the control, the HA biofilm and the DA biofilms (Fig. 5). The analysis confirmed significant enrichment (PPI enrichment p-value: < 1.0e-16) of proteins associated with cytokine-mediated (red) and chemokine-mediated (green) signaling pathways, the inflammatory response (blue) and positive regulation of the inflammatory response (yellow). Compared to the control, keratinocytes exposed to the HA biofilms secreted mainly cytokines and chemokines associated with baseline regulation of the inflammatory response (Fig. 5a). In contrast, keratinocytes exposed to the DA biofilms exhibited a robust pro-inflammatory response with release of cytokines and chemokines that are highly connected to one another (Fig. 5b).

**Table 1:** Significant proteins with VIP_pred_ (variable of importance-predictive) > 1.0 and absolute p(corr) > 0.3 that discriminate the secretome of keratinocytes exposed to health-associated (HA) biofilms from controls (CON). The negative sign of p(corr) indicates higher concentrations in HA compared to CON

Proteins	VIP_pred_	*p*(corr)	Fold changes HA vs. CON	*P*-value Mann Whitney
FLT3L	1.76	-0.98	2.14	< 0.001
IL-15	1.75	-0.98	2.03	< 0.001
IL-1RA	1.74	-0.97	2.17	< 0.001
IL-1α	1.72	-0.96	2.3	< 0.001
M-CSF	1.70	-0.95	1.53	< 0.001
IL-18	1.63	-0.91	2.27	< 0.001
IP-10	1.63	0.91	0.10	< 0.001
TSLP	1.61	0.85	0.15	0.007
TARC	1.59	0.88	0.23	0.012
MCP-1	1.48	0.82	0.80	0.002
G-CSF	1.47	0.82	0.80	< 0.001
TPO	1.45	-0.84	3.71	0.001
MIF	1.43	-0.80	1.06	0.003
Eotaxin-2	1.41	0.78	0.60	0.002
TRAIL	1.36	-0.76	4.18	< 0.001
IFN-β	1.28	0.72	0.94	0.005
ENA-78	1.27	0.71	0.60	0.004
IL-12p70	1.27	0.71	0.37	0.006
IL-33	1.10	-0.89	1.93	0.011
IL-1β	1.09	-0.61	1.19	0.115
EPO	1.07	0.66	0.08	0.009
IL-17 A	1.04	0.58	0.78	0.036

**Table 2:** Significant proteins with VIPpred (variable of importance-predictive) > 1.0 and absolute p(corr) > 0.4 that discriminate the secretome of keratinocytes exposed to disease-associated (DA) biofilms from controls (CON). The negative sign of p(corr) indicates lower concentrations in DA compared to CON

Proteins	VIP_pred_	*p*(corr)	Fold changes DA vs. CON	*P*-value Mann Whitney
VEGF-A	1.58	0.99	1.77	0.001
MIP-3α	1.58	0.98	11.18	0.001
IL-1β	1.57	0.98	6.83	0.001
M-CSF	1.56	0.97	1.56	0.001
GRO-α	1.56	0.97	7.55	0.001
IL-8	1.56	0.97	12.83	0.001
IL-1α	1.54	0.96	1.84	0.001
TNF-α	1.52	0.95	4.94	0.001
IL-18	1.50	0.93	1.86	0.001
IL-15	1.44	0.90	1.37	0.001
MCP-1	1.44	0.90	2.33	0.001
ENA-78	1.44	0.89	2.36	0.001
FLT3L	1.43	0.89	1.29	0.001
G-CSF	1.40	0.87	5.72	0.001
GM-CSF	1.40	0.87	14.38	0.001
IL-17 C	1.37	0.89	10.87	0.004
IL-10	1.37	0.88	2.64	0.020
IL-6	1.35	0.84	6.58	0.001
IL-1RA	1.28	0.80	1.24	0.004
IL-12p70	1.23	0.77	2.06	0.003
IP-10	1.21	0.75	2.57	0.003
TRAIL	1.20	0.71	5.45	0.005
TARC	1.14	-0.71	0.66	0.005
IFN-γ	1.09	0.69	3.05	0.001
IL-2	1.03	0.61	2.58	0.130
MIF	1.03	0.64	1.05	0.011
IFN-α2a	1.01	0.64	1.38	0.104

**Table 3:** Significant proteins with VIPpred (variable of importance-predictive) > 1.0 and p(corr) > 0.4 that discriminate the secretome of keratinocytes exposed to health-associated (HA) biofilms from those exposed to disease-associated (DA) biofilms. The negative sign of p(corr) indicates higher concentrations in HA compared to DA

Proteins	VIP_pred_	*p*(corr)	Fold changes HA vs. DA	*P*-value Mann Whitney
FLT3L	1.57	-0.98	1.66	0.001
MIP-3α	1.56	0.97	0.07	0.001
IL-1β	1.55	0.97	0.17	0.001
IL-8	1.55	0.97	0.07	0.001
IL-15	1.54	-0.96	1.48	0.001
GRO-α	1.54	0.96	0.13	0.001
VEGF-A	1.54	0.96	0.56	0.001
IL-1RA	1.52	-0.95	1.76	0.001
TNF-α	1.52	0.95	0.19	0.001
ENA-78	1.51	0.94	0.26	0.001
MCP-1	1.47	0.92	0.34	0.001
IL-12p70	1.43	0.89	0.18	0.002
IP-10	1.41	0.88	0.04	0.001
GM-CSF	1.41	0.88	0.07	0.001
IL-10	1.41	0.88	0.27	0.016
G-CSF	1.40	0.88	0.14	0.001
MIP-5	1.37	-0.86	1.57	0.001
IL-6	1.36	0.85	0.10	0.001
Eotaxin-2	1.23	0.77	0.57	0.002
IL-1α	1.17	-0.73	1.25	0.004
IL-17 C	1.15	0.77	0.08	0.040
MIP-1α	1.13	0.74	0.31	0.060
TSLP	1.10	0.78	0.12	0.008
YKL-40	1.06	0.66	0.65	0.021
SDF-1alpha	1.04	0.68	0.69	0.042
IL-17 F	1.03	-0.83	4.65	0.180
IFN-α2a	1.01	0.63	0.49	0.009

### Immunomodulatory effects of co-culture supernatants

**Fig. 6:**
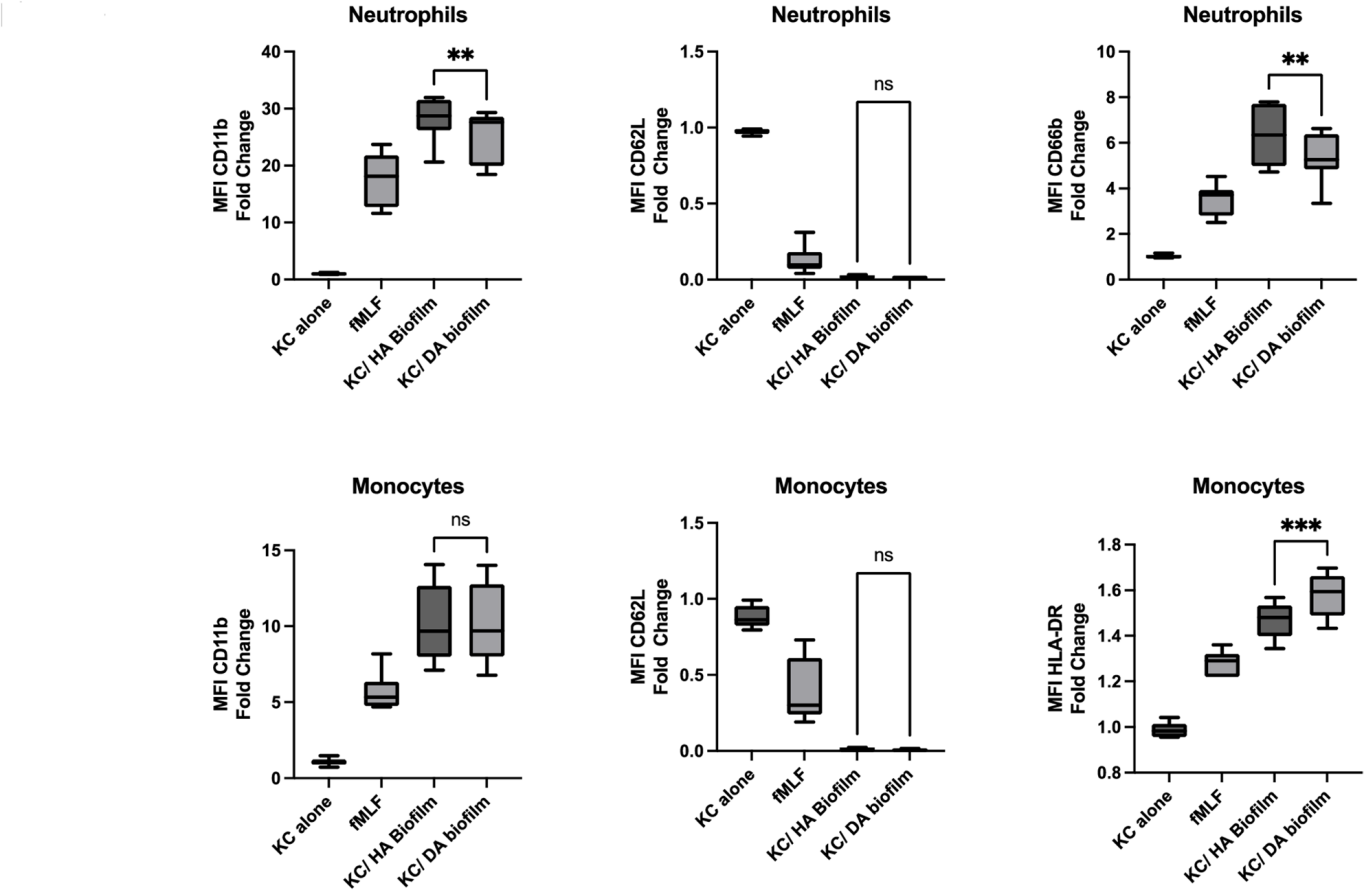
Effects of co-culture supernatants on the activation of leucocytes. Citrated whole blood from *n* = 7 independent donors was stimulated with HEPES (to determine baseline levels), 1 µM fMLF, or supernatants from keratinocytes exposed to HA biofilms, DA biofilms or buffer for 15 min at 37 °C. The median fluorescent intensities (MFIs) of the following activation markers were determined via flow cytometry: CD11b and CD62L on neutrophils and monocytes, CD66b on neutrophils and HLA-DR on monocytes. Data are shown as the fold change relative to the baseline value. Statistical analysis was performed via Student’s paired t test; ns = not significant, ** *P* < 0.01, *** *P* < 0.001

The effects of the co-culture medium on the activation of monocytes and neutrophils were investigated via flow cytometry. As expected, the positive control, N-formylmethionine-leucyl-phenylalanine (fMLF), activated both monocytes and neutrophils, whereas the medium from keratinocytes not exposed to biofilms (control) had no effect on either cell type. Compared with the positive control agonist fMLF, media from keratinocytes exposed to both the HA biofilm and the DA biofilm mediated stronger activation. Both biofilms resulted in significant upregulation of CD11b in the monocyte and neutrophil populations, with 10- to 20-fold increases in the MFI above the background (Fig. 6). In the case of neutrophil CD11b, significantly less activation was mediated by DA biofilms than HA biofilms (*p* < 0.005). CD62L is downregulated on both monocytes and neutrophils in response to activation, and both the HA and the DA biofilms significantly downregulated CD62L, with no significant differences observed in the level of downregulation between stimuli (Fig. 6). CD66b is released to the surface of activated neutrophils. Both biofilms stimulated CD66b release; however, the magnitude of the response generated by the DA biofilms was significantly lower than that generated by the HA biofilms (*p* < 0.005). HLA-DR is upregulated on the surface of activated monocytes. Both biofilms stimulated HLA-DR expression; however, the effects of the DA biofilms were significantly greater than those of the HA biofilms (*p* < 0.001). Collectively, these results demonstrate a strong inflammatory response from both monocytes and neutrophils exposed to medium from keratinocytes. Differential responses occur for the two leucocyte subsets investigated, whereby DA biofilms mediate more activation of monocytes and less activation of neutrophils.

## Discussion

Periodontal disease is a multifactorial condition involving dysregulated inflammation in periodontal tissues. The bacterial communities in the oral microbiome adapt to a changing environment, resulting in the selection of species that thrive in the protein-rich environment of the inflamed sulcus [14, 28]. Single pathogen studies have described mechanisms involved in the modulation of the keratinocyte barrier and/or immunomodulatory effects on leukocytes recruited from the blood to the mucosa. In this study, we developed a model of keratinocyte effector function in periodontitis that mimics an important aspect of the disease: the presence of complex, multispecies biofilms of bacteria. Although the model does not encompass the full range of bacterial species present in vivo, we successfully generated stable and viable biofilm communities over 7 days under anaerobic conditions containing species found in the periodontal pocket in health and disease [11, 14]. The bacterial products released from these communities diffuse into the keratinocyte medium and mediate the release of cytokines, chemokines and growth factors, and this complex co-culture medium mediates the activation of monocytes and neutrophils in downstream assays. Although this probably closely reflects the situation in vivo, one limitation of this experimental set-up is that co-culture material combines both the biofilm factors and the keratinocyte products, making it impossible to distinguish the origin of downstream effector molecules. The addition of keratinocyte-free controls could have helped to distinguish the direct effects of bacterial products on immune cells from effects mediated by keratinocyte-derived products alone. Short-term exposure to both HA and DA biofilms stimulated keratinocyte responses, but distinct profiles of mediators were enriched for the DA biofilms, which may contribute to the differential neutrophil and monocyte activation determined for these communities.

As expected, bacterial products from both HA and DA biofilms mediated keratinocyte activation. Some cytokines, including IL-15, IL-1RA, TPO and TRAIL, associated with immune surveillance and regulation were found at relatively high levels in response to HA biofilms, but overall, a robust pro-inflammatory cytokine response was not observed. In general, many well-established pro-inflammatory cytokines are released from keratinocytes exposed to DA biofilms. Some of these, such as IL-1β, TNF-α, and IL-6, have pleotrophic, pro-inflammatory effects and have previously been reported to be upregulated in periodontal disease, where they contribute to multi-factorial aspects of disease pathogenesis, such as the activation of epithelial cells, fibroblasts and osteoclasts that collaborate to mediate inflammation and the repair and remodelling of periodontal tissue [29, 30]. IL-17 C is a member of the IL-17 family of cytokines that is primarily released from activated epithelial cells acting as an important mediator of mucosal immune responses during the acute response to infection. IL-17 C can, for example, amplify the tissue response by stimulating the release of other IL-17 family cytokines from local immune cells, including IL-17 A which is an important driver of neutrophil activation and effector functions [31]. It reportedly enhances pro-inflammatory cytokine release in the presence of TNF-α. Furthermore, important chemokines for monocytes and neutrophils, including IL-8, GRO-α, MCP-1, ENA-78, and MIP3-α, were enriched in material from cells stimulated with DA biofilms. In particular, GRO-α and Il-8 upregulation in response to DA biofilms likely reflects the contribution of keratinocytes to the regulation of neutrophil recruitment and function. Our findings are in agreement with previous reports that oral keratinocytes and human gingival tissue display distinct profiles of cytokine upregulation when exposed to DA or HA consortia of oral bacteria [22, 32]. Collectively, these chemokines result in the recruitment of leukocytes into the tissue, which is a hallmark of periodontitis. As disease progresses, infiltrating cells switch from the early dominance of neutrophils to increased accumulation of macrophages and lymphocytes [29].

Our flow cytometry-based assays of leucocyte activation demonstrated that material derived from keratinocytes stimulated with both HA and DA biofilms strongly promoted leucocyte activation. This likely reflects both the downstream effects of the cytokines and chemokines derived from keratinocyte activation and the direct effects of bacterial products that diffuse into the keratinocyte medium. The robust activation of neutrophils and monocytes via both HA and DA biofilms highlights the importance of bacterial-derived products in co-culture media and mimics the pathogenesis of disease, where bacterial products rather than bacteria penetrate the keratinocyte barrier. Both CD11b and CD62L are involved in the transmigration of leukocytes from the blood into the tissue. An equivalent level of CD62L downregulation and CD11b upregulation was observed on monocytes exposed to material from both HA and DA biofilms, indicating that monocyte transmigration was not differentially affected in our experimental set-up. Differential expression of HLA-DR on monocytes, which is a marker of antigen uptake and processing, was observed. Compared with HA biofilms, DA biofilms significantly increased the level of HLA-DR on monocytes. HLA-DR has been reported to be decreased on circulating monocytes in periodontitis, reflecting an immunosuppressive phenotype [33]. In contrast to the effects on monocytes, the upregulation of both CD11b and CD66b on the cell surface was significantly lower in neutrophils exposed to DA biofilms than in those exposed to HA biofilms. These findings suggest that both transmigration and granule release are diminished under these circumstances. Neutrophils are primary sentinel cells that patrol the periodontium and protect the mucosal barrier. Neutrophils increase in the tissue during disease, and while this reflects a robust immune defence strategy, it is well established that this response becomes progressively dysregulated and destructive [34, 35]. Our findings suggest that complex multispecies DA biofilms are particularly detrimental to both the activation and recruitment of neutrophils.

Proteolytic activity was significantly greater in the DA biofilms than in the control biofilms, which confirms that an important attribute of bacteria associated with disease is maintained in our multi-species biofilms for up to 7 days. These proteases, particularly gingipains from *P. gingivalis*, have previously been described to degrade immune mediators and cytokines [36, 37]. This does not seem to have impacted our ability to detect cytokines in the material derived from keratinocytes stimulated with both HA and DA biofilms. Gingipains have previously been reported to mediate PAR receptor activation [24, 38], which may contribute to the activation of keratinocytes or direct activation of leucocytes in our experimental models.

Adaptation of the oral microbiome to gingival inflammation is a significant trigger of periodontitis in susceptible individuals. This disease-associated community then drives inflammation, and tissue damage is primarily mediated by this dysregulated inflammation. Here, we exploit an in vitro model to elucidate host responses to complex multi-species biofilms that are associated with either health or disease. The increased understanding of the dysregulated immune response mediated by HA compared to DA biofilms in our study provides insights into potential targets that may help to rebalance the inflammatory response and counteract destructive inflammation as an adjunct to periodontal treatment.

## Supplementary Information

Supplementary Material 1.

## Data Availability

The datasets supporting the conclusions of this article are included within the article and its additional files.

## Abbreviations

EGF: Epidermal growth factor
MALDI: TOF–matrix–assisted laser desorption/ionization–time of flight
