# Supplementary material for: Immunomodulatory effects of eubiotic and dysbiotic multi-species biofilms on oral keratinocytes
Source: BMC Oral Health. 2025 Dec 29;26:203. doi: 10.1186/s12903-025-07576-w (PMC12859963; doi:10.1186/s12903-025-07576-w)
Supplement: Supplementary file 1 — Supplementary Material 1. [file 12903_2025_7576_MOESM1_ESM.pdf]

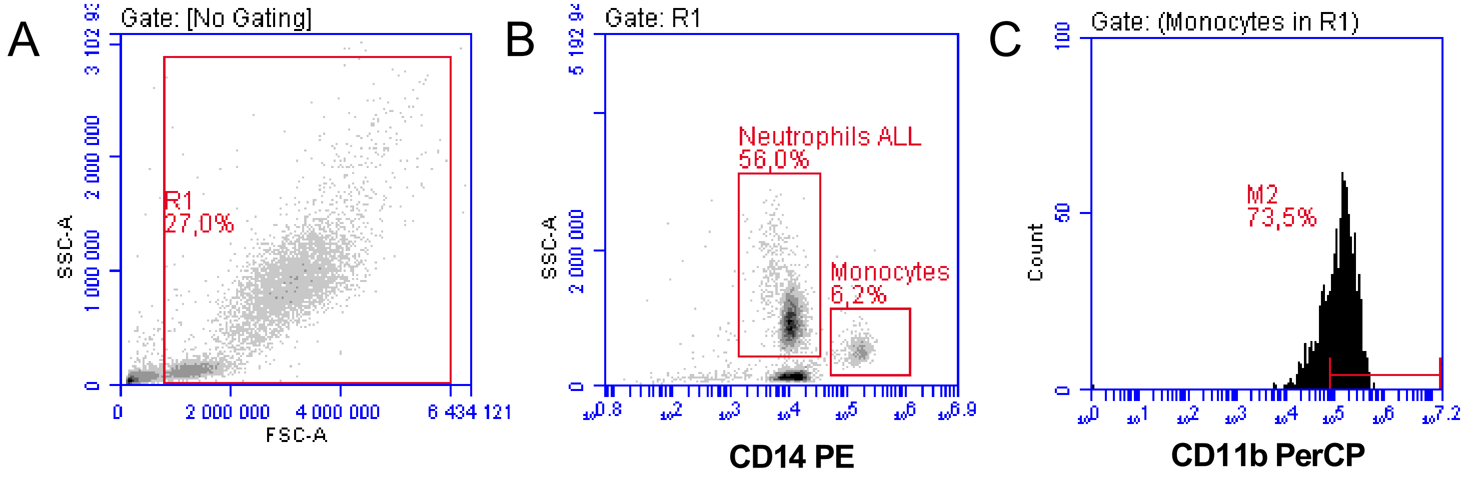

Supplementary Figure 1. Representative gating strategies and histograms for whole blood. **(A)** Leukocytes were gated using a linear scale FSC-A/SSC-A scatter plot with a FSC-H threshold of 150,000 to exclude red blood cell debris. **(B)** Neutrophils and monocytes were gated based on a CD14-PE/SSC-A scatter plot. (**C**)The median fluorescence intensity (MFI) of neutrophil and monocyte activation (CD11b-PE-Cy5) was determined based on histogram plots.
